# Use of a Liquid Supplement Containing 2 Human Milk Oligosaccharides: The First Double-Blind, Randomized, Controlled Trial in Pre-term Infants

**DOI:** 10.3389/fped.2022.858380

**Published:** 2022-04-25

**Authors:** Jean-Michel Hascoët, Marie Chevallier, Catherine Gire, Roselyne Brat, Jean-Christophe Rozé, Karine Norbert, Yipu Chen, Mickaël Hartweg, Claude Billeaud

**Affiliations:** ^1^Maternite Regionale Universitaire A. Pinard, Nancy, France; ^2^Hopital Couple Enfant, CHU de Grenoble Alpes, Grenoble, France; ^3^Hôpital Nord Chemin des Bourrely, Marseille, France; ^4^CHR Orléans, Orléans, France; ^5^Hôpital Femme Enfant Adolescent Néonatologie et Réanimation Pédiatrique, Nantes, France; ^6^Centre Hospitalier de Pau, Pau, France; ^7^Nestlé Product Technology Center-Nutrition, Vevey, Switzerland; ^8^Nestlé Clinical Development Unit, Lausanne, Switzerland; ^9^CHU de Bordeaux CIC, Hôpital des Enfants, Bordeaux, France

**Keywords:** pre-term, human milk oligosaccharides (HMOs), time to full enteral feeding, growth, nutrition, supplement

## Abstract

There is growing evidence supporting the benefit of human milk oligosaccharides (HMOs) on reducing risk of illnesses and improving immune function in newborn infants, but evidence in pre-term infants is lacking. This randomized, double-blind, placebo-controlled trial (NCT03607942) of pre-term infants evaluated the effects of HMO supplementation on feeding tolerance, growth, and safety in 7 neonatal units in France. Pre-term infants (27–33 weeks' gestation, birth weight <1,700 g) were randomized early after birth to receive HMO supplement (*n* = 43) [2′-fucosyllactose (2′FL) and lacto-*N*-neotetraose (LNnT) in a 10:1 ratio (0.374 g/kg body weight/day)] or an isocaloric placebo (*n* = 43) consisting of only glucose (0.140 g/kg/day) until discharge from the neonatal unit. Anthropometric *z*-scores were calculated using Fenton growth standards. Primary outcome was feeding tolerance, measured by non-inferiority (NI) in days to reach full enteral feeding (FEF) from birth in HMO vs. placebo group (NI margin = 4+ days). Mean number of days on intervention prior to FEF was 8.9 and 10.3 days in HMO and placebo, respectively. Non-inferiority in time to reach FEF in HMO (vs. placebo) was achieved [LS mean difference (95% CI) = −2.16 (−5.33, 1.00); upper bound of 95% CI < NI margin] in full analysis set and similar for per protocol. Adjusted mean time to reach FEF from birth was 2 days shorter in HMO (12.2) vs. placebo (14.3), although not statistically significant (*p* = 0.177). There was no difference in weight-for-age *z*-scores between groups throughout the FEF period until discharge. Length-for-age *z*-scores were higher in HMO at FEF day 14 [0.29 (0.02, 0.56), *p* = 0.037] and 21 [0.31 (0.02, 0.61), *p* = 0.037]. Head circumference-for-age *z*-score was higher in HMO vs. placebo at discharge [0.42 (0.12, 0.71), *p* = 0.007]. Occurrence of adverse events (AEs) was similar in both groups and relatively common in this population, whereas 2.3 and 14.3%, respectively, experienced investigator-confirmed, related AEs. HMO supplementation is safe and well-tolerated in pre-term infants. After 9 days of supplementation, the HMO group reached FEF 2 days earlier vs. placebo, although the difference was not statistically significant. In addition, HMO supplementation supports early postnatal growth, which may have a positive impact on long-term growth and developmental outcomes.

## Introduction

Pre-term birth is a major risk factor for neonatal sepsis and many other comorbidities (1). The current standard of care to treat infections in pre-term infants is with antibiotics upon the initial signs of risk, yet early prophylactic antibiotic usage has been found to cause an imbalance in the microbial population of the infant gut, which can lead to downstream complications (2). Prebiotics and probiotics promote the colonization of beneficial microbiota (3–6) and maybe a promising approach to prevent infections in pre-term infants (7, 8). Human milk oligosaccharides (HMOs) act as prebiotics and represent the third largest solid component in human milk after lactose and lipids (9–12). Breast milk from mothers who delivered premature infants contains approximately 9–23 g/L HMOs comprising more than 100 different known structures (9), and HMOs have been shown to prevent infection through multiple mechanisms, including aiding in the development of the immune system, binding of pathogens and toxins to prevent their uptake in the gut, enhancing the epithelial barrier function of the gut, and promoting the colonization of beneficial bacteria (11, 13–15).

Observational cohort studies have suggested reduced risk of illness or reduced mortality associated with specific HMOs in breast milk (2, 16–19). In pre-term infants, selective HMO such as disialyllacto-*N*-tetraose (DSLNT) has been found to be protective of necrotizing enterocolitis (NEC) (20, 21). There are only a few clinical studies to date, but two growth and safety clinical trials examining HMOs showed age-appropriate infant growth as well as a trend toward improved immune parameters (22) and lower illness incidence (23). However, since these trials were conducted in term infants, information on gastrointestinal (GI) tolerance outcomes that are specifically of interest in the pre-term population, such as time to reach full enteral feeding (FEF), is not available. Preclinical data in pre-term animal models have shown that different HMOs may confer benefit in pathology scores, incidence of NEC, and improving intestinal perfusion (24, 25). Collectively, these findings suggest plausible mechanisms by which HMOs may be beneficial for pre-term infants who are especially vulnerable to many illnesses.

There has not been an established consensus on the effect of HMOs on pre-term infant growth (26). However, it has been speculated that HMOs can have an effect on infant growth and development through multiple mechanisms, including directly on epithelial cell responses in the gut (27–30), through systemic effects after being absorbed into the blood stream (31–33), or in ways mediated by the gut microbiota (34–36). HMOs resist degradation in the small intestines and remain relatively intact until they reach the colon, where specific HMOs serve as substrates for microbes and help to shape the gut microbiome. Select microbiota patterns have been associated with alterations in energy harvest, influencing growth (34, 37, 38). Individual HMOs exhibit structure-function properties, where their chemical makeup helps to define different roles on infant health and development. As HMO supplementation has never been tested clinically among pre-term infants, the primary objective of this intervention trial was to establish safety and tolerance of HMO supplementation in pre-term infants. We also aimed to test the potential benefits of early supplementation in the initial period after birth when feeding is gradually advancing. During this critical period, pre-term infants are not receiving maximal levels of nutrients or bioactive components from their feeding to facilitate catch-up growth and healthy physical development. We hypothesized that HMO supplementation starting early after birth would confer developmental and overall health benefits to pre-term infants.

## Materials and Methods

This was a prospective, randomized, double-blind, controlled trial comparing a dietary supplement containing two HMOs to a placebo. The study was conducted at 7 neonatal units in France. Clinically stable (defined as not receiving any invasive respiratory support, absence of open ductus arteriosus requiring treatment, has not experienced early-onset sepsis or severe intrauterine growth retardation at birth and currently not using vasopressive drugs to support hemodynamics), pre-term infants born between 27 and 32 weeks of gestational age (GA), with birth weight < 1,700 g, and younger than 7 days of age were enrolled into the study. Infants were excluded if they were receiving ongoing prophylactic antifungal therapies or experiencing early-onset sepsis or liver failure prior to enrollment.

### Study Products

The experimental product was a liquid supplement containing 2 specific HMOs [2′-fucosyllactose (2′FL) and lacto-*N*-neotetraose (LNnT)] in a 10:1 ratio (0.340 and 0.034 g/kg body weight/day, respectively) equally distributed into 3 portions per day. The control product was a liquid placebo containing only glucose (0.140 g/kg body weight/day) matched to the HMO supplement with regard to energy content and comparable in color, odor, and viscosity, also equally distributed into 3 portions per day. The HMO and placebo products were blinded to both study staff and participants. The osmolalities of the HMO and placebo products were 275 and 240 mOsm/kg, respectively, which is very close to or meeting the range reported for human milk (279–297 mOsm/kg) (39). The energy content for both the HMO and placebo products was 22 kcal/100 ml.

### Study Design

Infants were randomized to receive either HMO supplement or placebo, with dosage computed each day according to each infant's daily weight. Both products were delivered in a sterile solution and given three times a day *via* syringe directly into the mouth or by enteral tube before feeding. Administration started as soon as 24 h of trophic feeding was possible but within 7 days of birth until discharge from the neonatal unit. Permitted dietary intakes during the supplementation period included human milk [either mother's own milk (MOM) or donor human milk (DHM)], human milk fortifier (HMF), and pre-term infant formula. Pre-term infant formula was only allowed after 1,500–1,800 g of body weight was reached as per the feeding protocol at the study sites. There were no restrictions on any specific type of HMF or pre-term formula, including all brands that were routinely used at the neonatal unit.

The study comprised three distinct periods, namely, pre-full enteral feeding (pre-FEF), FEF, and post-discharge follow-up until 24 months of corrected age (CA). The pre-FEF period began on the first day of product administration and lasted until the first day of FEF, hence, duration of the pre-FEF period varied by subject. FEF, defined as a minimal enteral intake of 150 ml/kg/day without parenteral nutrition, continued until discharge. Product administration ended on the day of discharge. There is a follow-up study monitoring the post-discharge follow-up period of these infants consisting of additional study visits at 2-, 12-, 18-, and 24-month CA. The results from the follow-up study are not presented here.

### Outcomes

The primary outcome of interest was time to FEF, which was a safety and potential efficacy end point. Secondary outcomes of interest within the neonatal unit period were anthropometric measures and associated *z*-scores, GI tolerance, and incidence and severity of all adverse events (AE). Anthropometric measures included weight, length, head circumference (HC), as well as gains in each anthropometric measure. Infants were weighed without diapers on calibrated electronic weighing scales (Baby Scale 727, Seca, Semur-en-Auxois, France) and with measurements recorded to the nearest 1 g. Recumbent length was measured with a calibrated pediatric length board (Measuring Board 417, Seca, Semur-en_auxois, France) by at least two trained examiners and recorded to the nearest 0.5 cm. HC was measured using a standard pediatric measurement tape (Measuring Tape 212, Seca, Semur-en-Auxois, France) by locating the maximum circumference of the head and recorded to the nearest 0.1 cm. Corresponding *z*-scores were computed according to the commonly used intrauterine-based Fenton growth standards (40). GI tolerance measures included volume of gastric residuals, duration of parenteral feeding, stool frequency, and stool consistency. Stool consistency was measured on a validated, pictorial, 5-point scale (1 = watery, 2 = runny, 3 = mushy soft, 4 = formed, 5 = hard) for every stool passed using a GI Tolerance and Symptoms diary. Additional intake data collected during hospitalization included number of missed feedings and types and amount of milk consumed from MOM, DHM, and infant formula. Both GI tolerance and intake data were reported daily by investigators from pre-FEF day 1 until FEF day 1, and for three consecutive days prior to FEF days 7, 14, 21, and finally at discharge. AEs were monitored throughout the study and were classified by system organ class (SOC) (41) and preferred term using MedDRA version 20.0.

### Statistical Analyses

Summary statistics were computed for infant demographics, baseline characteristics, treatment exposure, GI symptoms, and AEs. Sample size was calculated based on non-inferior testing of the primary outcome, which was the number of days from birth to reach FEF in the HMO supplement vs. placebo group. The non-inferiority margin was defined as ≤ +4 days, which was based on a large observational study conducted from 2011 to 2014 comparing time to reach FEF between hospital with limited access to human milk and other sites with predominantly human milk feeding (42). The upper end of the 95% CI of the treatment difference was 17.8 days, and based on the US Food and Drug Administration-recommended method (43, 44), it is prudent to use a conservative estimation of the minimum effect set at 25% of the upper boundary of the 95% CI, leading to a statistically justified non-inferiority margin of +4 days. As shorter time to reach enteral feeding is more ideal, non-inferiority margin is an indication of the highest tolerated difference in time to reach enteral feeding that we can allow, which will still be deemed to be safe clinically in regard to GI tolerance of pre-term infants. With an overall type I error of 5 and 80% power, the number of subjects to be included in the per protocol set (PPS) per group was 30. The non-inclusion rate from enrollment to PPS was assumed to be ~30%, resulting in a total of 43 infants per group to be enrolled. Major protocol deviations that exclude subjects from PPS include wrong dose administrations, violations of inclusion/exclusion criteria, and suspension of investigational product due to AEs.

Analyses regarding the primary outcome were done in a hierarchical manner to control for the experiment-wise false-positive rate. First, time to FEF was analyzed for non-inferiority on PPS, then full analysis set (45). FAS included all the subjects who have measurements for the primary outcome of time to reach FEF. After non-inferiority had been achieved on both datasets, superiority analysis would be conducted on FAS. Time to FEF was modeled using analysis of covariance (ANCOVA) adjusting for birth weight, study center, and sex of the infant. Group differences in weight, length and HC gains, and associated *z*-score changes were modeled using mixed effects models on the intention-to-treat (ITT) analysis set, adjusting for each respective measure at baseline, as well as study center, sex, and mode of delivery. ITT analysis set included all infants randomly assigned to one of the two treatment groups who are administered at least one feeding of the study formula and who have their birth date and date of reaching FEF (150 ml/kg/day and discontinuation of parenteral nutrition) recorded. Group differences were calculated for each timepoint and for overall effect over the entire study period. GI tolerance analyses were performed on the ITT analysis set and presented for each individual study period. Prior to FEF, GI tolerance measures were calculated using the average of daily estimates. After FEF, GI tolerance measures were calculated at weekly intervals based on the assumption that the data captured in 3-day diaries are approximately representative of the patterns over the entire week after FEF had been reached. Daily mean gastric residual volumes (in ml/kg) were computed as the sum of reported gastric residual volumes (in ml) divided by the number of incidences linked to the visit and by the infant weight at the visit. Stool frequency was described as the number of stools linked to the visit divided by the number of days. Stool consistency linked to the visit was calculated as the arithmetic mean of all recorded consistencies during all days linked to the visit. Group differences in all the GI tolerance measures were tested using ANCOVA model adjusting for birth weight, center, and sex. Incidence of AEs was compared between randomized groups on the safety analysis set (SAF) using logistic regression correcting for birth weight, GA, and center, or Fisher's exact test if logistic regression models did not converge. Tests of differences between groups for all the outcomes were 2-sided with alpha = 0.05. All analyses were conducted using SAS 9.4.

## Results

**Figure 1:**
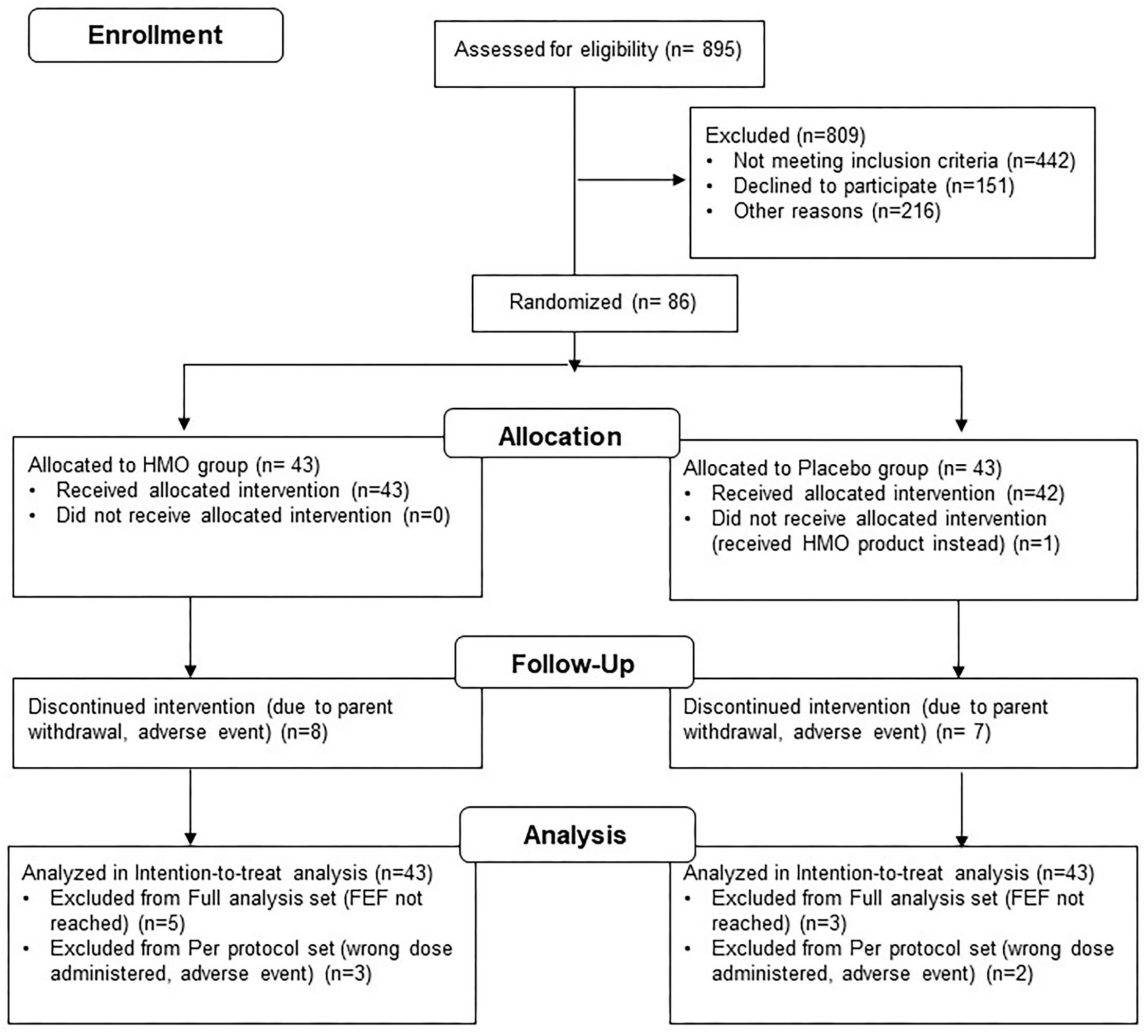
Subject disposition.

There were 86 infants randomized and included in the ITT population (HMO, *n* = 43; placebo, *n* = 43). One subject was randomized to placebo but actually received HMO, thus the SAF population included 44 and 42 subjects in the HMO and placebo groups, respectively. A total of 7 and 8 infants experienced premature discontinuation from the trial in the HMO group (due to investigator decision, *n* = 1; without any explanation, *n* = 2; parents' decision, *n* = 4; due to AE, *n* = 1) and placebo group (due to investigator decision, *n* = 1; without any explanation, *n* = 1; parents' decision, *n* = 3; due to AE, *n* = 2), respectively. A total of 71 infants completed the study treatment through the discharge visit, and 78 infants were included in the FAS (Figure 1). After consideration of major protocol deviations, 73 infants were included in the PPS (HMO, *n* = 35; placebo, *n* = 38).

Infant demographics and baseline measures are summarized in Table 1. Approximately half of the infants were of male gender (57%), and the majority of them were of Caucasian descent (86%), with mean (SD) chronological age at enrollment of 6.3 (1.3) and 6.2 (1.4) days in the HMO and placebo groups, respectively. At birth, infants had a mean GA of 29 weeks + 5.2 days in HMO and 30 weeks + 1.5 days in the placebo group. Approximately 4.7 and 9.3% of infants in the HMO and placebo groups, respectively, were very pre-term (GA <28 weeks). The percentage of very low birth weight (<1,500 g) was 86% and 91% in HMO and placebo arms, respectively. In the ITT population, during the pre-FEF period (baseline to FEF day 1), the mean number of days of intervention was 8.9 (SD 5.2, range 2–28) in the HMO and 10.3 (SD 10.0, range 2–66) in the placebo group (*p* = 0.16) (data not shown), while in the FEF period, the mean number of days of intervention was 35.5 (SD 17.3, range 4–66) in the HMO group and 33.1 (SD 26.9, range 0–127) in the placebo group (*p* = 0.62). During the pre-FEF period, the mean proportions of MOM, DHM, and infant formula received from enteral feeding were 41.9 vs. 42.9% for MOM, 58.1 vs. 54.8% for DHM, and 0 vs. 2.4% for infant formula in the HMO vs. placebo groups, respectively. The proportion of infant formula increased during the FEF period such that by FEF day 21 the proportions of MOM, DHM, and infant formula in the HMO vs. placebo groups were 57.1 vs. 33.3% for MOM, 27.3 vs. 54.5% for DHM, and 9.5 vs. 18.2% for infant formula, respectively. The mean intake rates for each type of feeding are summarized in Table 2.

**Table 1:** Infant characteristics by study arm, ITT population (*N* = 86).

	**HMO ***N*** = 43**	**Placebo ***N*** = 43**
**Sex,** ***n*** **(%)**		
Male	18 (41.9)	19 (44.2)
Female	25 (58.1)	24 (55.8)
**Ethnicity**, ***n*** **(%)**		
Asian	1 (2.3)	0 (0.0)
Black	4 (9.3)	1 (2.3)
White	36 (83.7)	38 (88.4)
Other	2 (4.7)	4 (9.3)
Chronological age (days) at enrollment, mean (SD)	6.3 (1.3)	6.2 (1.4)
Gestational age (weeks + days) at birth, mean (SD)	29 w + 5.2 d (1 w + 2.5 d)	30 w + 1.5 d (1 w + 2.9 d)
**Mode of delivery**, ***n*** **(%)**		
Vaginal	15 (34.9)	18 (41.9)
Cesarean	28 (65.1)	25 (58.1)
Birth weight-for-age Z-score, mean (SD)	−0.37 (0.72)	−0.56 (0.76)
Birth length-for-age Z-score, mean (SD)	−0.42 (0.98)	−0.46 (0.91)
Birth head circumference-for-age Z-score, mean (SD)	−0.44 (0.91)	−0.27 (1.14)
Extremely low birth weight (<1,000 g)	21%	19%
Small for gestational age (birth weight z-score < -1.28)	9.3%	20.9%

**Table 2:** Types of milk intake.

	**HMO group (mL/kg/day) ***N*** = 43**	**Placebo group (mL/kg/day) ***N*** = 43**
**Pre-FEF period**		
Mother's own milk (MOM)	41.29 ± 47.57	38.35 ± 42.24
Donor human milk (DHM)	62.18 ± 52.08	63.52 ± 49.29
Infant formula (IF)	1.45 ± 6.66	4.36 ± 28.24
**FEF day 7**		
MOM	69.43 ± 64.97	82.13 ± 74.01
DHM	56.60 ± 64.22	58.73 ± 72.08
IF	6.05 ± 29.28	1.78 ± 6.89
**FEF day 14**		
MOM	56.60 ± 64.22	58.73 ± 72.08
DHM	44.03 ± 65.06	46.34 ± 67.05
IF	13.36 ± 40.32	7.5 ± 25.42
**FEF day 21**		
MOM	61.01 ± 70.71	40.46 ± 58.90
DHM	30.61 ± 55.48	56.02 ± 57.78
IF	8.21 ± 26.32	18.77 ± 36.93
**Hospital discharge**		
MOM	13.20 ± 38.07	0
DHM	2.69 ± 13.44	0
IF	21.84 ± 69.60	52.26 ± 126.13

*Mean estimates ± SD are presented*.

### Time to FEF

Adjusted mean time to FEF (Table 3) was 12.15 (9.50, 14.81) days in the HMO and 14.32 (11.71, 16.92) days in the placebo group. Non-inferiority was well-demonstrated; the adjusted mean difference was −2.16 days (95% CI: −5.33, 1.00, *p* < 0.001) for non-inferiority analysis in FAS and −1.95 days (95% CI: −5.27, 1.37, *p* < 0.001) in PPS. Infants in the HMO group (vs. placebo) reached FEF faster by 2 days after 9 days of intervention in the pre-FEF period, although the difference was not statistically significant (*p* = 0.177 for superiority analysis in FAS).

**Table 3:** Time to reach full enteral feeding (FEF) by study arm, FAS, and PP populations.

	**HMO**		**Placebo**		**Adjusted mean treatment difference^a^ HMO—Placebo**	
	**FAS (***n*** = 38)**	**PP (***n*** = 35)**	**FAS (***n*** = 40)**	**PP (***n*** = 38)**	**FAS**	**PP**
Time from birth to FEF (days), LS means (95% CI)	12.15 (9.50, 14.81)	11.91 (9.14, 14.69)	14.32 (11.71, 16.92)	13.86 (11.12, 16.60)	−2.16* (−5.33, 1.00)	−1.95* (−5.27, 1.37)
Min, Max	7, 30	7, 30	5, 70	5, 70	–	–
Q1, Q3	9, 14	9, 14	8.5, 15.5	8, 14	–	–

a *Adjusted estimates are based on an ANCOVA model adjusting for birth weight, study site, and sex of infant*.

* *p < 0.001 for noninferiority analysis (i.e., upper bound of 95% CI <4 + days) in both FAS and PPS*.

### Anthropometrics

**Figure 2:**
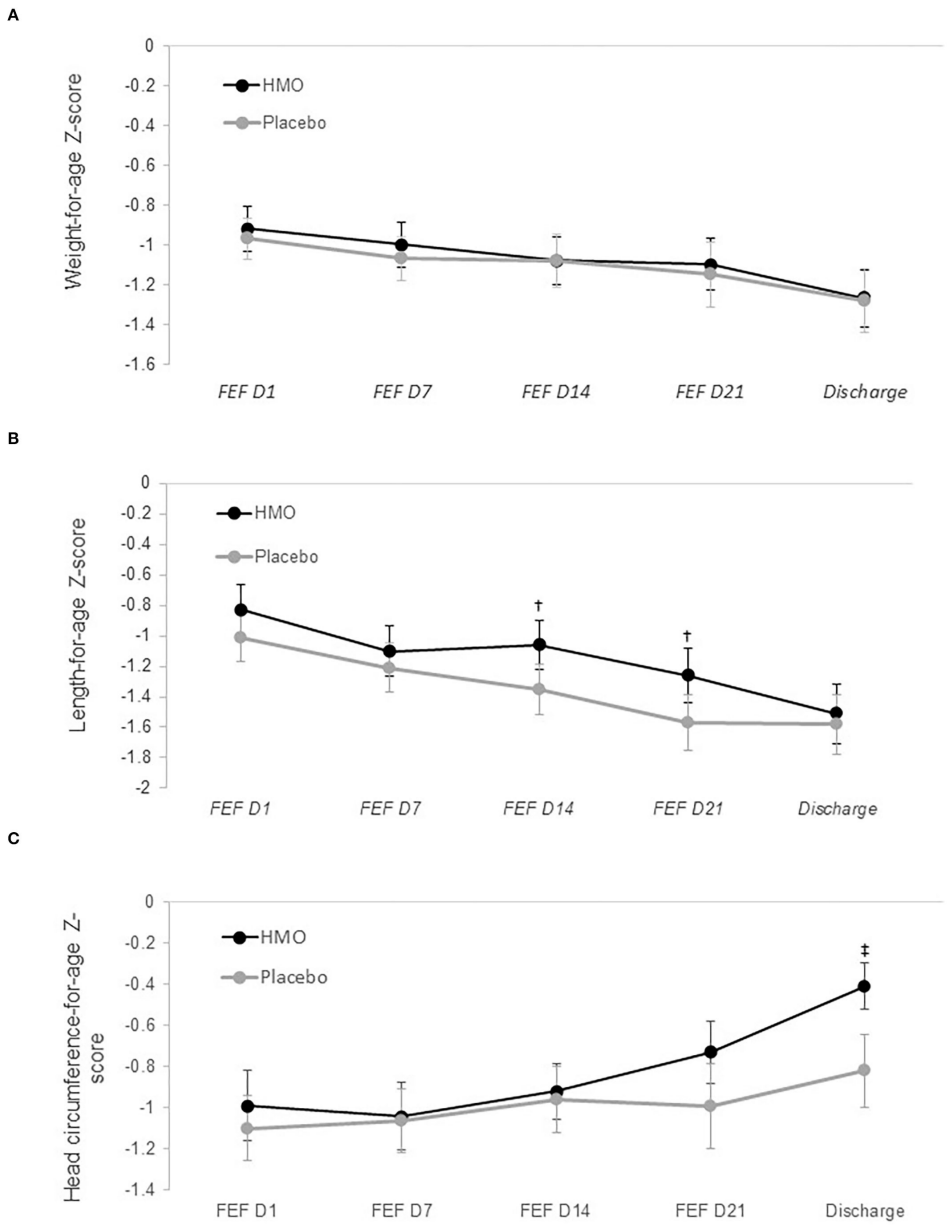
Adjusted mean anthropometric *z*-scores for weight **(A)**, length **(B)**, and head circumference **(C)** by randomized group. Circles (and whiskers) in graph indicate LS means (±1 SE), which were derived from mixed model with repeated measures, adjusting for measure at birth, measure at baseline, study center, sex, and mode of delivery. FEF, full enteral feeding. †*p* = 0.037; ‡*p* = 0.007.

Adjusted mean *z*-scores for weight, length, and HC from FEF day 1 until discharge are shown in Figure 2. Overall, weight gain (g/day) from baseline to discharge was similar between groups with adjusted mean difference between HMO and placebo of −0.98 g/day (95% CI: −3.89, 1.93; *p* = 0.50). Weight gain rates steadily increased in both groups over the study period (data not shown). Adjusted mean rates ranged from 18.5 g/day at FEF day 1 to 26.0 g/day at discharge in the HMO group and 20.2 g/day at FEF day 1 to 26.2 g/day in the placebo group. Weight-for-age *z*-score (WAZ) was also similar between groups with an adjusted overall mean difference of 0.03 (95% CI: −0.13, 0.20; *p* = 0.68). Length gains in cm/week were not statistically significantly different between groups throughout the FEF period (overall treatment difference 0.09, 95% CI: −0.14, 0.33; *p* = 0.42), while length-for-age *z*-score (LAZ) was significantly higher in the HMO group compared to placebo at FEF days 14 and 21 with mean *z*-score differences of 0.29 (95% CI: 0.02, 0.56; *p* = 0.037) and 0.31 (95% CI: 0.02, 0.61; *p* = 0.037), respectively. Adjusted mean length gains increased over the FEF period in both groups, reaching greatest gains of 0.95 and 0.80 cm/week in the HMO and placebo groups, respectively, on FEF day 14, and sustained rates at 0.92 and 0.77 cm/week in the HMO and placebo groups, respectively, on FEF day 21. HC gain in cm/week was not significantly different between the groups over the FEF period (adjusted mean difference of 0.11, 95% CI: −0.08, 0.30; *p* = 0.243), but mean HC gain (cm/week) in the HMO group was borderline significantly greater than in the placebo group at FEF day 21 (adjusted mean difference 0.14, 95% CI: −0.01, 0.28; *p* = 0.059) and at discharge (adjusted mean difference 0.12, 95% CI: −0.01, 0.25; *p* = 0.060). In addition, mean gains in HC followed similar patterns as weight and length, where the gains progressively increased during the FEF period, reaching greatest gains of 0.93 and 0.80 cm/week in the HMO and placebo groups, respectively, at FEF day 21. By discharge, HC gain was sustained at 0.92 and 0.79 cm/week in the HMO and placebo groups, respectively. Head circumference-for-age *z*-score (HCAZ) was similar between groups early in the FEF period but showed steady increases in the HMO group starting at FEF day 21 with significantly greater scores in HMO at discharge by 0.42 SD (95% CI: 0.12, 0.71; *p* = 0.007).

### GI Tolerance

Gastric residual volume was low in both groups, with daily means ranging from 0.05 to 3.03 ml/kg/day in the HMO group and 0.07–3.26 ml/kg/day in the placebo group from pre-FEF period to discharge. Daily mean stool frequencies ranged from 3.09 to 4.17 stools/day in HMO and from 3.11 to 4.20 stools/day in placebo from pre-FEF period to discharge. There were no significant differences between the HMO and placebo groups in stool frequency during any weekly intervals except from FEF day 1–day 7, where the HMO group (vs. placebo) had slightly lower stool frequency (adjusted mean difference −0.54 stool/day, 95% CI: −1.07, −0.01; *p* = 0.046). Mean daily stool consistency ranged 2.97–3.11 (HMO) and 2.91–3.19 (placebo) from the pre-FEF period to discharge, falling approximately in the “mushy soft” category on the consistency scale at all timepoints.

### Adverse Events

A total of 40 (90.9%) and 36 (85.7%) subjects experienced an AE in the HMO and placebo groups, respectively, over the study period. A total of 7 (15.9%) and 2 (4.8%) subjects experienced serious AEs (SAEs) in the HMO and placebo groups, all distributed within GI disorders, General disorders and administration site conditions, and respiratory, thoracic, and mediastinal disorders SOC categories. Among all the AEs, 1 (2.3%) and 6 (14.3%) subjects in the HMO and placebo groups, respectively, experienced AEs that were assessed by the study investigator to be related to the study intervention, and only 1 SAE (NEC) in the placebo group was considered to be related to the study intervention. A total of 4 SAEs led to withdrawal of the study product (2 each in the HMO and placebo groups or 4.5 and 4.8%, respectively). Illnesses and infections from baseline to discharge are summarized in Table 4. There were no significant differences in occurrences of illness and infection AEs between groups.

**Table 4:** Incidence of infant illnesses and infections from baseline to discharge.

**Selected illnesses (by preferred terms)**	**HMO (***N*** = 43)**	**Placebo (***N*** = 43)**	**Odds ratio [95% CI] or Fisher's Exact** ***p*****-value^a^**
Necrotizing enterocolitis	3 (7.9%)	2 (5.0%)	*p* = 0.67
Late onset sepsis	0 (0.0%)	2 (5.0%)	*p* = 0.49
Bronchopulmonary dysplasia	10 (23.3%)	13 (30.2%)	0.58 [0.19; 1.77]
Gram-positive/negative bacterial and fungal sepsis	11 (25.6%)	9 (20.9%)	*p* = 0.80
Mortality	0 (0.0%)	0 (0.0%)	*na*
GI and urinary tract infections	0 (0.0%)	2 (4.7%)	*p* = 0.49
Retinopathy of pre-maturity	3 (7.0%)	2 (4.7%)	*p* > 0.99
Intraventricular hemorrhage	3 (7.0%)	3 (7.0%)	*p* > 0.99
Periventricular leukomalacia	1 (2.3%)	0 (0.0%)	*p* > 0.99
Neonatal inflammatory response syndrome	0 (0.0%)	0 (0.0%)	*na*
Alteration of liver function	1 (2.3%)	1 (2.3%)	*p* > 0.99

a *For all group comparisons of adverse events, logistic regression models were used correcting for birth weight, GA, and center. If the model did not converge, Fisher's exact test was used to compare between groups the proportion of subjects experiencing the AE. No models converged with the exception of bronchopulmonary dysplasia*.

## Discussion

This is the first trial to demonstrate that an HMO supplement specifically for pre-term infants is safe, well-tolerated, and associated with improved growth patterns in length and HC. The primary outcome of this study, time to FEF, was selected because it is a reliable indicator of feeding tolerance. Feeding tolerance is an important clinical outcome for pre-term infants as it reflects the efficiency of nutrient metabolism and any risk factors for morbidities such as NEC. While this was the first study to examine time to FEF in human milk-fed pre-term infants receiving HMOs as a prebiotic supplement, a 2010 trial by Modi et al. examined time to FEF in infants randomized to receive either a standard formula or a formula supplemented with prebiotic galacto-oligosaccharides (GOS) and fructooligosaccharides (FOS), in a dosage to make up any shortfall of MOM. The median time to FEF was longer in the supplemented group by 1 day but was not statistically significant (46). There are several other studies examining the effect of probiotics on time to FEF. In a meta-analysis examining the effects of probiotics on time to FEF in pre-term, very-low-birth-weight infants according to feeding type (human milk vs. formula), Aceti et al. found a reduction of 3 days in time to FEF among 5 studies of human milk-fed infants receiving probiotics compared to those receiving placebo; only two studies of formula-fed infants were identified and neither demonstrated any difference in time to FEF between the probiotic and control groups (47). We found a reduction of time to FEF by 2 days with HMO supplement when compared to the placebo, after just 9 days of intervention. However, this difference did not reach statistical significance. This was expected since the trial was powered to detect a larger effect (4 days). However, clinicians may consider this difference to be clinically relevant in certain situations.

*In vitro* studies have also offered valuable insights into the possible mechanisms by which the two HMOs 2′FL and LNnT might improve time to FEF in pre-term infants. Both 2′FL and LNnT have been found *in vitro* to enhance fermentation of synbiotic mutualistic microbiota, as evidenced by significantly increased amounts of known metabolites of beneficial bacteria such as lactate and short-chain fatty acids (48). Supplementation of *in vitro* cultures with 2′FL promoted growth and metabolic activity of beneficial *Bifidobacteria* and inhibited the pathological *Campylobacter, Campylobacter jejuni* and *Escherichia coli* (49–52). In addition to conferring benefits to the microbiota composition and metabolism, 2′FL also improved immune function by diminishing insults from *E. coli* by quenching proinflammatory cytokines and reduced food allergy symptoms in mouse models (53, 54). There is limited preclinical work in pre-term animal models, but Autran et al. compared NEC pathology scores among neonatal rats receiving only formula, an HMO blend (10 mg/ml), GOS (8 mg/ml), sialylated GOS (0.4 mg/ml), or 2′FL (2 mg/ml) and demonstrated that 2′FL alone significantly improved pathology scores when compared with the formula-only and GOS-only groups (24). Another study examined the efficacy of 2′FL in neonatal mice induced with NEC and found that the administration of 0.25 mg/g body weight of 2′FL once daily significantly reduced the severity of NEC compared with mice receiving only a standard formula, and further, the 2′FL-supplemented mice experienced restored intestinal perfusion to a level more comparable to that of breastfed mice and significantly different from that seen in the standard formula-fed group (25). A third preclinical animal study found that an HMO cocktail, including LNnT fed to pre-term piglets, tended to improve NEC resistance after 11 days (55). Overall, the available clinical and preclinical research done with mostly formula-fed subjects elucidates mechanisms by which HMOs may improve intestinal integrity and immunity, supporting the improvement of GI tolerance as measured by time to FEF that was observed in this study. The shorter time to reach FEF has been found to be associated with cognitive function in a follow-up study of pre-term infants who had experienced NEC in early neonatal life (56), and in other studies pre-term infants who had shorter time to reach FEF also had shorter time to hospital discharge (57), reduced feeding interruption and episodes of feeding intolerance (58, 59), and better weight gain (58).

A more striking finding of this study was the moderate but significant improvement in length and HC by discharge. Notably, we found that the HMO group had a higher value of 0.3 SD in LAZ by FEF day 14, which persisted until day 21. Moreover, the HMO group also had a higher value of 0.4 SD in HCAZ by discharge. This effect on HC slowly increased over the FEF period but became most apparent and significant at discharge. The effects that we observed on length and HC are clinically meaningful as HMO supplementation was seen to influence growth outcomes beyond infant weight. Less attention has been paid to linear stunting in spite of the relationship between linear growth and organ development and promotion of cellular differentiation during the early neonatal period (60). Improved linear growth in the absence of accelerated weight gain is thus indicative of lean body mass gain in this study.

Several published studies have documented the link between quality of growth, specifically in length and HC status, and infant development. An observational study conducted in the US among 62 low-birth-weight infants who were appropriate for GA found that length *z*-score at discharge and 4-month CA were negatively associated with days of oxygen, days of steroids, days to FEF, and days of antibiotics and inpatient caloric deficit (61). In addition, even after adjustment of weight and HC *z*-scores, length *z*-score at discharge was positively associated with Bayley speech scores while 4- and 12-month CA length *z*-scores were positively associated with Bayley cognitive scores. Another large cohort study conducted in the US with 613 pre-term infants born at <33 weeks' GA revealed that head growth between first week of birth and term was positively associated with Bayley cognitive scores at 18-month CA (45). Other studies have found similar results. At 3-month CA, head size was a significant predictor of neurodevelopmental delay at 12 and 24 months of CA among a group of 538 Austrian pre-term infants born <32 weeks GA (62). This effect persisted until 5-year CA in the long-term follow-up (63). Moreover, the associations between head *z*-scores and cognitive scores at 12- and 24-month CA became weaker when compared to the 3-month data (62), indicating that early neonatal growth may have a long-lasting programming effect even if there is an eventual growth normalization. In a review on long-term growth and general health for the smallest or most immature infants, Roberts and Cheong pointed out that early growth has a significant influence on final height, which is related to overall health and self-esteem in prematurely born adolescents (64). Despite improved clinical management of pre-term infants over the past decade, postnatal extrauterine growth retardation during the neonatal period remains to be an issue in premature neonates. Zhao et al. showed that 37% of 691 premature neonates, eutrophic at birth, presented extrauterine growth retardation at discharge (65). Thus, an intervention allowing even a moderate improvement in growth parameters is beneficial for these infants.

To the best of our knowledge, there is no study evaluating the effect of HMOs on pre-term infant growth. There exists only limited data among undernourished population. For instance, in a rural Gambian population, 3′SL levels in human milk from 4 to 20 weeks of lactation have been found to be positively correlated with WAZ at 20 weeks of age (66). Similarly, Lacto-N-fucopentaose I (LNPF I) I was positively correlated with HCAZ at 20 weeks. In the same study, it was observed that LNFP I was higher in milk samples of infants who did not have any sick days, and the authors alluded to a potential immune benefit of LNFP I supporting growth by sparing resources that would otherwise be spent fighting off infection (66). Another study involving Malawian infants and mother dyads at 6-month postpartum found that 2′FL and LNFP I are the two fucosylated HMOs most discriminatory for infant growth (67). Similar to LNFP I, 2′FL is also an α(1, 2) fucosylated structure. We saw in this study the effect of HMOs on growth parameters among pre-term infants, which is consistent with available literature studying undernourished populations.

This first interventional trial studying the effect of HMO supplementation pre-term infants pioneers the way forward for adoption of these innovative ingredients in the neonatal unit. The HMOs selected in this clinical trial are reflective of the two HMOs with the most sufficient safety data in term infants and pre-term preclinical models since HMOs have not been clinically tested before in pre-term infants. The ratio of the product tested is also reflective of the natural abundance of 2′FL and LNnT found in pre-term milk samples (68). Strengths of this trial include the adherence to local feeding and nutrition guidelines of each neonatal unit, which resulted in the infants receiving a variety of feeding regimens, including MOM, DHM, and pre-term infant formula. This increases the generalizability of study results since the feeding practice and protocol are similar to most NICUs worldwide. This landmark study tested a unique supplementation regimen adapted to the daily weight of pre-term infants, ensuring that safety and efficacy could be captured with an adjustable dose of HMOs to mimic natural milk intake. With respect to safety, the subjects were exposed to the study intervention for a significant amount of time even after reaching FEF (mean of >30 days in both groups), which provides the safety assurance HMO supplementation for an extended period of time. There were some limitations. To prevent a selection bias with regard to the primary outcome of interest, infants were included only when they were clinically stable and generally healthy. In addition, infants who presented with early-onset sepsis or severe intrauterine growth retardation at birth were excluded. Given the potential benefits we saw on feeding tolerance and growth, a more vulnerable population of pre-term infants could have benefited more from the study intervention. In addition, Gabrielli et al. (9) showed that there is a 40% higher level of 2′FL in the colostrum than that of mature milk, suggesting that this prebiotic may have a priming effect. Therefore, one might speculate that a mean postnatal age of 7 days might have already been too late with regard to the physiology of these compounds. Thus, the effect of the intervention may have been reduced in the study. Achieving early supplementation of HMOs is critical since the pre-term infant is not receiving maximal enteral feeding during this time frame prior to FEF.

In conclusion, a pre-term infant supplement with the HMOs 2′FL and LNnT was shown to be safe and well-tolerated among pre-term infants. When given shortly after birth, the HMO supplement was associated with improved growth parameters in length and HC during the hospitalization period.

## Data Availability Statement

The datasets presented in this article are not readily available because further sharing of data was not included in the informed consent form signed by study participants. Requests to access the datasets should be directed to yipu.chen@nestle.com.

## Ethics Statement

The studies involving human participants were reviewed and approved by Comite de Protection des Personnes Est-III. Written informed consent to participate in this study was provided by the participants' legal guardian/next of kin.

## Author Contributions

J-MH, MC, CG, RB, J-CR, KN, and CB supervised the study by providing essential medical oversight at the study sites. MH was involved in all the statistical analysis. J-MH and YC took the lead in writing the manuscript. All authors contributed to the final version of the manuscript and provided critical feedback in the study design and interpretation of the data throughout the study.

## Funding

This study was sponsored by Société des Produits Nestlé S.A.

## Conflict of Interest

This study received funding from Société des Produits Nestlé S.A. The funder had the following involvement with the study: interpretation of data, writing of this article, and decision to submit it for publication. The authors declare that the research was conducted in the absence of any commercial or financial relationships that could be construed as a potential conflict of interest.

## Publisher's Note

All claims expressed in this article are solely those of the authors and do not necessarily represent those of their affiliated organizations, or those of the publisher, the editors and the reviewers. Any product that may be evaluated in this article, or claim that may be made by its manufacturer, is not guaranteed or endorsed by the publisher.

## Abbreviations

HMOs: human milk oligosaccharides
disialyllacto-*N*-tetraose (DSLNT)
GI: gastrointestinal
FEF: full enteral feeding
NEC: necrotizing enterocolitis
2′FL: 2′-fucosyllactose
LNnT: lacto-*N*-neotetraose
MOM: mother's own milk
DHM: donor human milk
HMF: human milk fortifier
FEF: full enteral feeding
CA: corrected age
AE: adverse events
SOC: system organ class
PT: preferred term
PPS: per protocol set
FAS: full analysis set
ITT: intention to treat
SAF: safety analysis set
SD: standard deviation
GA: gestational age
SAE: serious AEs
HC, head circumference WAZ: weight-for-age *z*-score
LAZ: length-for-age *z*-score
HCAZ: head circumference-for-age *z*-score.

## References

[1] Behrman RE Butler AS. Institute of Medicine (US) Committee on Understanding Premature Birth and AssuringHealthOutcomes. Mortality and acute complications in preterm infants. In: Preterm Birth: Causes, Consequences, and Prevention. Washington, DC: National Academies Press (2007). 20669423

[2] Greenwood C Morrow AL Lagomarcino AJ Altaye M Taft DH Yu Z. Early empiric antibiotic use in preterm infants is associated with lower bacterial diversity and higher relative abundance of enterobacter. J Pediatr. (2014) 165:23–9. 10.1016/j.jpeds.2014.01.010 24529620 PMC4074569

[3] Mugambi MN Musekiwa A Lombard M Young T Blaauw R. Probiotics, prebiotics infant formula use in preterm or low birth weight infants: a systematic review. Nutr J. (2012) 11:58. 10.1186/1475-2891-11-58 22928998 PMC3487753

[4] Kapiki A Costalos C Oikonomidou C Triantafyllidou A Loukatou E Pertrohilou V. The effect of a fructo-oligosaccharide supplemented formula on gut flora of preterm infants. Early Hum Dev. (2007) 83:335–9. 10.1016/j.earlhumdev.2006.07.003 16978805

[5] Fanaro S Boehm G Garssen J Knol J Mosca F Stahl B. Galacto-oligosaccharides and long-chain fructo-oligosaccharides as prebiotics in infant formulas: a review. Acta Paediatr. (2005) 94:22–6. 10.1111/j.1651-2227.2005.tb02150.x 16214761

[6] Olsen R Greisen G Schrøder M Brok J. Prophylactic probiotics for preterm infants: a systematic review and meta-analysis of observational studies. Neonatology. (2016) 109:105–12. 10.1159/000441274 26624488

[7] Luoto R Ruuskanen O Waris M Kalliomäki M Salminen S Isolauri E. Prebiotic and probiotic supplementation prevents rhinovirus infections in preterm infants: a randomized, placebo-controlled trial. J Allergy Clin Immunol. (2014) 133:405–13. 10.1016/j.jaci.2013.08.020 24131826 PMC7112326

[8] Weichert S Schroten H Adam R. The role of prebiotics and probiotics in prevention and treatment of childhood infectious diseases. Pediatr Infect Dis J. (2012) 31:859–62. 10.1097/INF.0b013e3182620e52 22801095

[9] Gabrielli O Zampini L Galeazzi T Padella L Santoro L Peila C. Preterm milk oligosaccharides during the first month of lactation. Pediatrics. (2011) 128:e1520–31. 10.1542/peds.2011-1206 22123889

[10] Hale T Hartmann PE. Textbook of Human Lactation. Springer (2007).

[11] Niñonuevo MR Lebrilla CB. Mass spectrometric methods for analysis of oligosaccharides in human milk. Nutr Rev. (2009) 67 Suppl 2:S216–26. 10.1111/j.1753-4887.2009.00243.x 19906226

[12] Zivkovic AM German JB Lebrilla CB Mills DA. Human milk glycobiome and its impact on the infant gastrointestinal microbiota. Proc Natl Acad Sci USA. (2011) 108 (Suppl. 1):4653–8. 10.1073/pnas.1000083107 20679197 PMC3063602

[13] Kulinich A Liu L. Human milk oligosaccharides: the role in the fine-tuning of innate immune responses. Carbohydr Res. (2016) 432:62–70. 10.1016/j.carres.2016.07.009 27448325

[14] Lewis ZT Totten SM Smilowitz JT Popovic M Parker E Lemay DG. Maternal fucosyltransferase 2 status affects the gut bifidobacterial communities of breastfed infants. Microbiome. (2015) 3:13. 10.1186/s40168-015-0071-z 25922665 PMC4412032

[15] Bode L. The functional biology of human milk oligosaccharides. Early Hum Dev. (2015) 91:619–22. 10.1016/j.earlhumdev.2015.09.001 26375354

[16] Celik IH Demirel G Canpolat FE Dilmen U. Reduced plasma citrulline levels in low birth weight infants with necrotizing enterocolitis. J Clin Lab Anal. (2013) 27:328–32. 10.1002/jcla.21607 23852794 PMC6807425

[17] Mantis NJ Rol N Corthésy B. Secretory IgA's complex roles in immunity and mucosal homeostasis in the gut. Mucosal Immunol. (2011) 4:603–11. 10.1038/mi.2011.41 21975936 PMC3774538

[18] Pang T Leach ST Katz T Day AS Ooi CY. Fecal biomarkers of intestinal health and disease in children. Front Pediatr. (2014) 2:6. 10.3389/fped.2014.00006 24479111 PMC3904282

[19] Pezzilli R Barassi A Morselli-Labate AM Fantini L Tomassetti P Campana D. Fecal calprotectin and elastase 1 determinations in patients with pancreatic diseases: a possible link between pancreatic insufficiency and intestinal inflammation. J Gastroenterol. (2007) 42:754–60. 10.1007/s00535-007-2086-0 17876545

[20] Autran CA Kellman BP Kim JH Asztalos E Blood AB Spence ECH. Human milk oligosaccharide composition predicts risk of necrotising enterocolitis in preterm infants. Gut. (2018) 67:1064–70. 10.1136/gutjnl-2016-312819 28381523

[21] Masi AC Embleton ND Lamb CA Young G Granger CL Najera J. Human milk oligosaccharide DSLNT and gut microbiome in preterm infants predicts necrotising enterocolitis. Gut. (2021) 70:2273–82. 10.1136/gutjnl-2020-322771 33328245 PMC9231288

[22] Goehring KC Marriage BJ Oliver JS Wilder JA Barrett EG Buck RH. Similar to those who are breastfed, infants fed a formula containing 2'-fucosyllactose have lower inflammatory cytokines in a randomized controlled trial. J Nutr. (2016) 146:2559–66. 10.3945/jn.116.236919 27798337

[23] Marriage BJ Buck RH Goehring KC Oliver JS Williams JA. Infants fed a lower calorie formula with 2'FL show growth and 2'FL uptake like breast-fed infants. J Pediatr Gastroenterol Nutr. (2015) 61:649–58. 10.1097/MPG.0000000000000889 26154029 PMC4645963

[24] Autran CA Schoterman MH Jantscher-Krenn E Kamerling JP Bode L. Sialylated galacto-oligosaccharides and 2'-fucosyllactose reduce necrotising enterocolitis in neonatal rats. Br J Nutr. (2016) 116:294–9. 10.1017/S0007114516002038 27212112

[25] Good M Sodhi CP Yamaguchi Y Jia H Lu P Fulton WB. The human milk oligosaccharide 2'-fucosyllactose attenuates the severity of experimental necrotising enterocolitis by enhancing mesenteric perfusion in the neonatal intestine. Br J Nutr. (2016) 116:1175–87. 10.1017/S0007114516002944 27609061 PMC5124125

[26] Binia A Lavalle L Chen C Austin S Agosti M Al-Jashi I. Human milk oligosaccharides, infant growth, and adiposity over the first 4 months of lactation. Pediatr Res. (2021) 90:684–93. 10.1038/s41390-020-01328-y 33446921

[27] Holscher HD Davis SR Tappenden KA. Human milk oligosaccharides influence maturation of human intestinal Caco-2Bbe and HT-29 cell lines. J Nutr. (2014) 144:586–91. 10.3945/jn.113.189704 24572036

[28] Kuntz S Rudloff S Kunz C. Oligosaccharides from human milk influence growth-related characteristics of intestinally transformed and non-transformed intestinal cells. Br J Nutr. (2008) 99:462–71. 10.1017/S0007114507824068 17925055

[29] Chichlowski M De Lartigue G German JB Raybould HE Mills DA. Bifidobacteria isolated from infants and cultured on human milk oligosaccharides affect intestinal epithelial function. J Pediatr Gastroenterol Nutr. (2012) 55:321–7. 10.1097/MPG.0b013e31824fb899 22383026 PMC3381975

[30] Bode L Jantscher-Krenn E. Structure-function relationships of human milk oligosaccharides. Adv Nutr. (2012) 3:383s−91s. 10.3945/an.111.001404 22585916 PMC3649474

[31] Dotz V Rudloff S Meyer C Lochnit G Kunz C. Metabolic fate of neutral human milk oligosaccharides in exclusively breast-fed infants. Mol Nutr Food Res. (2015) 59:355–64. 10.1002/mnfr.201400160 25330044

[32] Ruhaak LR Stroble C Underwood MA Lebrilla CB. Detection of milk oligosaccharides in plasma of infants. Anal Bioanal Chem. (2014) 406:5775–84. 10.1007/s00216-014-8025-z 25059723 PMC4157097

[33] Goehring KC Kennedy AD Prieto PA Buck RH. Direct evidence for the presence of human milk oligosaccharides in the circulation of breastfed infants. PLoS ONE. (2014) 9:e101692. 10.1371/journal.pone.0101692 24999728 PMC4085000

[34] Turnbaugh PJ Ley RE Mahowald MA Magrini V Mardis ER Gordon JI. An obesity-associated gut microbiome with increased capacity for energy harvest. Nature. (2006) 444:1027–31. 10.1038/nature05414 17183312

[35] Subramanian S Blanton LV Frese SA Charbonneau M Mills DA Gordon JI. Cultivating healthy growth and nutrition through the gut microbiota. Cell. (2015) 161:36–48. 10.1016/j.cell.2015.03.013 25815983 PMC4440586

[36] Newburg DS Morelli L. Human milk and infant intestinal mucosal glycans guide succession of the neonatal intestinal microbiota. Pediatr Res. (2015) 77:115–20. 10.1038/pr.2014.178 25356747

[37] Eriksen KG Christensen SH Lind MV Michaelsen KF. Human milk composition and infant growth. Curr Opin Clin Nutr Met Care. (2018) 21:200–6. 10.1097/MCO.0000000000000466 29461264

[38] Ley RE Turnbaugh PJ Klein S Gordon JI. Microbial ecology: human gut microbes associated with obesity. Nature. (2006) 444:1022–3. 10.1038/4441022a 17183309

[39] De Curtis M Candusso M Pieltain C Rigo J. Effect of fortification on the osmolality of human milk. Arch Dis Childh Fetal Neonatal Ed. (1999) 81:F141–3. 10.1136/fn.81.2.F141 10448185 PMC1720981

[40] Fenton TR Kim JH. A systematic review and meta-analysis to revise the fenton growth chart for preterm infants. BMC Pediatr. (2013) 13:59. 10.1186/1471-2431-13-59 23601190 PMC3637477

[41] Société Française de Pédiatrie CdN. [Medium-chain triglyceride and their use in premature infants]. ArchFr Pediatr. (1993) 50:263–5. 8338422

[42] de Waard M Li Y Zhu Y Ayede AI Berrington J Bloomfield FH. Time to full enteral feeding for very low-birth-weight infants varies markedly among hospitals worldwide but may not be associated with incidence of necrotizing enterocolitis: the NEOMUNE-NeoNutriNet cohort study. J Parent Enteral Nutr. (2019) 43:658–67. 10.1002/jpen.1466 30465333 PMC6531355

[43] FDA. Non-Inferiority Clinical Trials to Establish Effectiveness: Guidance for Industry. Silver Springs, MD: FDA (2016).

[44] US Food and Drug Administration. Non-Inferiority Clinical Trials to Establish Effectiveness: Guidance for Industry. Silverspring, MD: US Food and Drug Adminstration (2016).

[45] Belfort MB Rifas-Shiman SL Sullivan T Collins CT McPhee AJ Ryan P. Infant growth before and after term: effects on neurodevelopment in preterm infants. Pediatrics. (2011) 128:e899–906. 10.1542/peds.2011-0282 21949135 PMC3182845

[46] Modi N Uthaya S Fell J Kulinskaya E. A randomized, double-blind, controlled trial of the effect of prebiotic oligosaccharides on enteral tolerance in preterm infants (ISRCTN77444690). Pediatr Res. (2010) 68:440–5. 10.1203/PDR.0b013e3181f1cd59 20639792

[47] Aceti A Gori D Barone G Callegari ML Fantini MP Indrio F. Probiotics and time to achieve full enteral feeding in human milk-fed and formula-fed preterm infants: systematic review and meta-analysis. Nutrients. (2016) 8:471. 10.3390/nu8080471 27483319 PMC4997384

[48] Vester Boler BM Rossoni Serao MC Faber TA Bauer LL Chow J Murphy MR. In vitro fermentation characteristics of select nondigestible oligosaccharides by infant fecal inocula. J Agric Food Chem. (2013) 61:2109–19. 10.1021/jf305056f 23379900

[49] Newburg DS Ruiz-Palacios GM Altaye M Chaturvedi P Meinzen-Derr J Guerrero Mde L. Innate protection conferred by fucosylated oligosaccharides of human milk against diarrhea in breastfed infants. Glycobiology. (2004) 14:253–63. 10.1093/glycob/cwh020 14638628

[50] Weichert S Jennewein S Hüfner E Weiss C Borkowski J Putze J. Bioengineered 2'-fucosyllactose and 3-fucosyllactose inhibit the adhesion of *Pseudomonas aeruginosa* and enteric pathogens to human intestinal and respiratory cell lines. Nutr Res. (2013) 33:831–8. 10.1016/j.nutres.2013.07.009 24074741

[51] Yu ZT Chen C Kling DE Liu B McCoy JM Merighi M. The principal fucosylated oligosaccharides of human milk exhibit prebiotic properties on cultured infant microbiota. Glycobiology. (2013) 23:169–77. 10.1093/glycob/cws138 23028202 PMC3531294

[52] Yu ZT Chen C Newburg DS. Utilization of major fucosylated and sialylated human milk oligosaccharides by isolated human gut microbes. Glycobiology. (2013) 23:1281–92. 10.1093/glycob/cwt065 24013960 PMC3796377

[53] Castillo-Courtade L Han S Lee S Mian FM Buck R Forsythe P. Attenuation of food allergy symptoms following treatment with human milk oligosaccharides in a mouse model. Allergy. (2015) 70:1091–102. 10.1111/all.12650 25966668

[54] He Y Liu S Kling DE Leone S Lawlor NT Huang Y. The human milk oligosaccharide 2'-fucosyllactose modulates CD14 expression in human enterocytes, thereby attenuating LPS-induced inflammation. Gut. (2016) 65:33–46. 10.1136/gutjnl-2014-307544 25431457

[55] Rasmussen SO Martin L Østergaard MV Rudloff S Roggenbuck M Nguyen DN. Human milk oligosaccharide effects on intestinal function and inflammation after preterm birth in pigs. J Nutr Biochem. (2017) 40:141–54. 10.1016/j.jnutbio.2016.10.011 27889684

[56] Kuik SJ den Heijer AE Mebius MJ Hulscher JBF Bos AF Kooi EMW. Time to full enteral feeding after necrotizing enterocolitis in preterm-born children is related to neurodevelopment at 2-3 years of age. Early Hum Dev. (2020) 147:105091. 10.1016/j.earlhumdev.2020.105091 32492527

[57] Kohler JA Perkins AM Bass WT. Human milk versus formula after gastroschisis repair: effects on time to full feeds and time to discharge. J Perinatol. (2013) 33:627–30. 10.1038/jp.2013.27 23519369

[58] Athalye-Jape G Deshpande G Rao S Patole S. Benefits of probiotics on enteral nutrition in preterm neonates: a systematic review. Ame J Clin Nutr. (2014) 100:1508–19. 10.3945/ajcn.114.092551 25411286

[59] Mohammadizadeh M Ghazinour M Iranpour R. Efficacy of prophylactic oral erythromycin to improve enteral feeding tolerance in preterm infants: a randomised controlled study. Singapore Med J. (2010) 51:952–6. 21221501

[60] Forbes GB. Relation of lean body mass to height in children and adolescents. Pediatr Res. (1972) 6:32–7. 10.1203/00006450-197201000-00005 5046969

[61] Ramel SE Demerath EW Gray HL Younge N Boys C Georgieff MK. The relationship of poor linear growth velocity with neonatal illness and two-year neurodevelopment in preterm infants. Neonatology. (2012) 102:19–24. 10.1159/000336127 22441508

[62] Neubauer V Griesmaier E Pehböck-Walser N Pupp-Peglow U Kiechl-Kohlendorfer U. Poor postnatal head growth in very preterm infants is associated with impaired neurodevelopment outcome. Acta Paediatr. (2013) 102:883–8. 10.1111/apa.12319 23772884

[63] Neubauer V Fuchs T Griesmaier E Kager K Pupp-Peglow U Kiechl-Kohlendorfer U. Poor postdischarge head growth is related to a 10% lower intelligence quotient in very preterm infants at the chronological age of five years. Acta Paediatr. (2016) 105:501–7. 10.1111/apa.13336 26792418

[64] Roberts G Cheong JL. Long-term growth and general health for the tiniest or most immature infants. Semin Fetal Neonatal Med. (2014) 19:118–24. 10.1016/j.siny.2013.11.003 24289903

[65] Zhao T Feng HM Caicike B Zhu YP. Investigation into the current situation and analysis of the factors influencing extrauterine growth retardation in preterm infants. Front Pediatr. (2021) 9:643387. 10.3389/fped.2021.643387 33996689 PMC8119632

[66] Davis JC Lewis ZT Krishnan S Bernstein RM Moore SE Prentice AM. Growth and morbidity of gambian infants are influenced by maternal milk oligosaccharides and infant gut microbiota. Sci Rep. (2017) 7:40466. 10.1038/srep40466 28079170 PMC5227965

[67] Charbonneau MR O'Donnell D Blanton LV Totten SM Davis JC Barratt MJ. Sialylated milk oligosaccharides promote microbiota-dependent growth in models of infant undernutrition. Cell. (2016) 164:859–71. 10.1016/j.cell.2016.01.024 26898329 PMC4793393

[68] Austin S De Castro CA Sprenger N Binia A Affolter M Garcia-Rodenas CL. Human milk oligosaccharides in the milk of mothers delivering term versus preterm infants. Nutrients. (2019) 11:1282. 10.3390/nu11061282 31195757 PMC6627155

